# Evaluation of the Impact of Storage Conditions on the Biogenic Amines Profile in Opened Wine Bottles

**DOI:** 10.3390/molecules23051130

**Published:** 2018-05-09

**Authors:** Justyna Płotka-Wasylka, Vasil Simeonov, Jacek Namieśnik

**Affiliations:** 1 Department of Analytical Chemistry, Faculty of Chemistry, Gdańsk University of Technology, 11/12 Narutowicza Street, 80-233 Gdańsk, Poland; chemanal@pg.edu.pl; 2 Faculty of Chemistry and Pharmacy, University of Sofia, 1 James Bourchier Blvd., Sofia 1126, Bulgaria; vsimeonov@chem.uni-sofia.bg

**Keywords:** biogenic amines, chemometric analysis, DLLME, GC-MS, storage conditions, stopper type

## Abstract

A survey of biogenic amine (BA) profiles in opened wine bottles has been established to monitor the level of biogenic amines (BAs) in opened bottles against time and other conditions. Bottles of red and white wine were submitted to different temperatures, stopper type (screw cap, cork), and use of vacuum devices. A total of six wines made from a variety of grapes were obtained from vineyards from regions across Poland. Dispersive liquid-liquid microextraction-gas chromatography-mass spectrometry (DLLME-GC-MS) procedure for BAs determination was validated and applied for wine sample analysis. The total content of BAs from the set of immediately opened wine samples ranged from 442 to 929 µg/L for white wines, and 669 to 2244 µg/L for red wines. The most abundant BAs in the analysed wines were histamine and putrescine. Considering the commercial availability of the analysed wines, there was no relationship between the presence of BAs in a given wine and their availability on the market. However, it was observed and confirmed by chemometric analysis that the different storage conditions employed in this experiment affect not only the BAs profile, but also the pH.

## 1. Introduction

Biogenic amines (BAs) are compounds which are naturally synthesized in microorganisms, animals, and plants and may be generally considered as a food hazard. Although there is not a threshold for these biomolecules in European legislation (except for histamine in fish and its products), many scientists are currently researching this area. BAs can influence the important physiological processes/functions in an organism, but the amount required is limited, thus, excess concentrations taken via food ingestion are reported to cause toxicological effects to the organisms [1]. BAs also have some beneficial contributions reported to be important to the taste of food. 

Biogenic amines are mainly produced by microbial decarboxylation of some amino acids, but also volatile amines can be formed by amination and transamination of aldehydes and ketones [2]. As BAs are stable compounds, once formed they are difficult to eliminate. The most common health effect of BAs is known as food poisoning and is implicated with different types of food products, mainly fish but also meat, cheese, and alcoholic beverages. The most important BAs occurring in food and beverages are histamine, β-phenylethylamine, cadaverine, putrescine, tyramine, serotonine, tryptamine, spermine, and spermidine. Although all of these are of high importance when present in food, histamine is the main causative biogenic amine to induce food poisoning in the majority of reported cases [3]. Moreover, some of the other BAs have been claimed to potentiate histamine food poisoning. In addition, BAs produced through the decomposition of food, including cadaverine and putrescine, or during processing (e.g., tyramine) are reported to have the potential to cause illness, even in the absence of histamine [3].

Alcohol is an inhibitor of monomine oxidase, thus the monitoring and control of BAs in fermented beverages, including wine, is very important for the health of consumers. In fact, the content of BAs in wine could also impact on commercial import and export procedures. Three possible origins of BAs in wine are reported [4]. BAs can be present in the must, can be produced by yeast during malolactic fermentation, or originate from the action of bacteria involved in the fermentation process. Other factors may also play an important role in the final concentration of BAs in wine, for example, nitrogenous fertilization, geographic location of grape, its variety, climatic conditions during growth, or the level of maturation may cause changes in the amino acids profile in grapes [5]. In addition, the concentration of amino acids may be changed by different pre-fermentation treatments such as clarification, crushing, or the duration of the maceration process [5]. On the other hand, it is also reported that conditions for BAs formation are generally related to factors affecting the growth of microorganisms that have decarboxylating activity and initiate this reaction in enzymes [3]. 

Other reaction-influencing factors include pH, temperature, oxygen content, and salt and sugar content. For example, it has been reported that decarboxylase activity of amino acids is stronger in an acidic environment [3]. Previously, the optimum pH for decarboxylating activity was suggested to be in the range 2.5–6.5, but currently is limited to 3.5–5.5. It is frequently reported that the quantitative production of BAs is time/temperature dependent. Thus, a faster amine production rate is evident with increasing temperature up to certain level while the production is minimum at low temperatures due to the inhibition of microbial growth and the reduction of enzyme activity [3]. It is reported, that optimum temperature for BA formation by mesophilic bacteria ranges between 20 °C and 37 °C, whereas BA production decreases above 40 °C and below 5 °C [6]. Since BAs in wine originate from many sources, this alcoholic beverage has specifically been studied throughout its different stages of elaboration and storage. Therefore, the concentration of BAs has been determined at various stages of wine production, starting from grapes [7] and musts [8,9,10] through the alcoholic and malolactic fermentation [10,11,12,13], aging in barrels or tanks [14,15] and storage in closed bottles [16,17,18]. However, reports focusing on the changes in BAs concentration in opened wine bottles are scarce. 

It is a popular problem that wine consumers many often drink wine several days after opening the wine bottle and sometimes the wine is kept at room temperature. Moreover, in the restaurant sector, wine is frequently stored in opened bottles. It is therefore important to monitor the level of BAs in opened bottles against time and other conditions. Therefore, this work is focused on evaluation of the profile of selected biogenic amines (histamine—HIS, cadaverine—CAD, putrescine—PUT, tyramine—TYR, tryptamine—TRYP, and 2-phenylethylamine—2-PE) in opened bottles of red and white wine exposed to different temperatures, types of stopper (screw cap, cork), and the use of vacuum devices. Even though information on BAs is currently not included in wine composition databases, information on their existence, distribution, concentration, and knowledge of existing relationships between BAs and other parameters is crucial and useful for the food industry, health professionals, and consumers [19].

## 2. Results and Discussion

The profiling of biogenic amine occurrence and their levels was evaluated in opened wine bottles over time. Wine bottles were stored under different conditions in terms of temperature and type of stopper. The monitoring of BA occurrence and levels was performed in just-opened bottles, seven days after opening and thirty days after opening. 

### 2.1. Biogenic Amines Profile in Just-Opened Bottles

Information on the concentration of BAs determined in the different wine samples by GC-MS technique are summarised in Table 1. Generally, red wines have higher amounts of BAs than white wines [5,20,21], but what was surprising was that the total concentration of BAs in the white wines originating from Solaris grapes was higher than those produced from Regent grapes (red wines). Therefore, the total content of BAs in white wines ranged from 442 to 929 µg/L, while in red wines ranged from 669 to 2244 µg/L in just-opened wine samples. 

In fact, red wines coming from Regent grapes have similar total BA content: 669 and 671 µg/L (both for commercial and non-commercial samples, respectively), while in those obtained from Frontenac grapes were about three times higher (2244 µg/L). White wines obtained from the same type of grapes (Solaris) had different total concentration of BAs. 

The most abundant BAs in the six analysed wines were histamine and putrescine, as expected (Table 1). However, in one non-commercial white wine sample made from 100% Solaris grapes, the concentration of tryptamine is two times higher than the concentration of putrescine. The content of all BAs in red wines obtained from Regent grapes was similar, so it can be concluded that they have similar profile of BAs. The wine produced from Frontenac grapes had a totally different characterisation with regard to BAs content. However, tyramine was under the limit of detection in all red wine samples. 

Considering white wine samples, there was no similarity in its characterisation of BAs profile. 

Considering the commercial availability of the analysed wines, there was no relationship between the presence of BAs in a given wine and their availability on the market.

### 2.2. Effect of the Storage Time and Conditions

Considering different storage conditions of opened wine bottles, slight changes were observed in the profile of BAs and pH (Table 1).

In the all red wines, the total amount of BAs showed a significant downward trend with time when storage was at room temperature. When samples were maintained at 4 °C, the total BA content also decreased, however, changes in concentration were small. The type of stopper impacted the concentration of all BAs. Those samples kept in cork-stoppered bottles showed a significant downward trend with time, while those kept under vacuum conditions did not show significant changes in the total concentrations of BAs. Samples kept in a screw-cap bottle also showed a downward trend with time, but these changes were not as big as for cork-stoppered samples. In all red wines, the same trend in changes of concentration in appropriate BAs was observed. These trends were as follows:

- 2-PE increased with time, but greater differences were visible between 7 and 30 days after opening, especially when wines were kept at room temperature;
- putrescine, tryptamine, and histamine decreased with time in all conditions, but these changes were more significant for histamine and putrescine when samples were maintained at room temperature;
- cadaverine content slightly decreased from the opening day to the seventh day, and then increased significantly from the seventh to the tenth day in all cases.

The changes in concentration of BAs maintained at 4 °C were so low that they do not affect the total concentrations. 

Like red wines, white wines show a clear and significant, but different trend in the total content of BAs. While higher concentrations of BAs were noted in red wines for just-opened bottles, in white wines, higher total concentrations of BAs were observed in samples seven days after opening under all storage conditions. The content of putrescine and cadaverine slightly increased over time, but these changes were not significant for refrigerated samples or those kept under vacuum. A significant increase in histamine concentrations from the opening day to seventh day was observed, while from seventh day to thirtieth day the concentration decreased significantly. The same trend was observed for 2-phenylethylamine and tyramine (in one sample, while in other tyramine was not detected) compared with red wines (Table 1). Tryptamine significantly increased among time in all conditions. 

Despite the fact that changes in concentration levels of BAs in white wine samples kept at 4 °C were smaller than those kept at room temperature, the total concentration of BAs was higher in these samples. A lower concentration of BAs was found in samples maintained at room temperature under vacuum.

**Figure 1:**
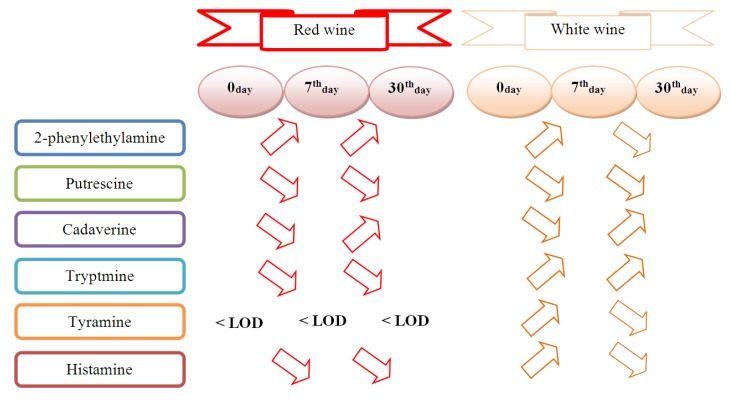
Schematic representation of the way BAs concentration changed over time.

In general, the evolution of BAs in the six analysed wines shows a clear common trend. It should be pointed out that the concentration of only one compound, namely putrescine, decreased in all wine samples, red or white. Other BAs concentrations differed between white and red wines (Figure 1). For example, in the case of histamine concentration, it decreased in red wines, while in white wines it was increased in first days then decreased from 7th to 30th day. 

Moreover, it was observed that the different storage conditions employed in this experiment affect not only the BAs profile, but also the pH. In red wines, the pH was higher in wines kept at room temperature than those kept at 4 °C. The pH differed with the type of stopper used at different temperatures, pH was higher when a screw cap was used than in the case of a cork stopper, but lower than pH of the samples under vacuum. 

In white wines, different stoppers did not show a clear common trend. 

### 2.3.Chemometric Assessment of Biogenic Amines Impact in Wines

Hierarchical and non-hierarchical cluster analyses were applied to a data set with different wines checked for the presence of six specific organic compounds. The major goal of the study was to reveal latent relationships between wine brands, the conditions for their storage, and their amine content. Altogether six wine brands were studied (marked as A, B, C, D, E, and F) for levels of 2-PE, PUT, CAD, TRYP, TYR, HIS, and, additionally, time of opening the sample bottles (after 0, 7, and 30 days) and type of stopper (cork, screw cap, and stopper by vacuum pump). Temperature of storage (room temperature and 4 °C) were checked in the experimentation. 

This is a typical multivariate problem and, therefore, the chemical data were treated and interpreted using multivariate analysis.

#### 2.3.1. Relationship Between Chemical Variables

Hierarchical and non-hierarchical cluster analyses for all six wine brands was performed, each input set having set dimensions [18 × 6]. The different conditions applied to a specific brand (temperature of storage, type of stopper, and time of opening) were involved as objects and the concentrations of the six amines as variables. It is important to note that in some cases not all six variables were used since some did not show any change with the variation of the experimental conditions and were actually not detected in the brand. This decreased the number of the variables used. 

Clustering of chemical compounds (only for Wine A all six compounds were used as variable, for D, E, F—five variables were available and for Wine B—only four) is presented in Table 2.

**Figure 2:**
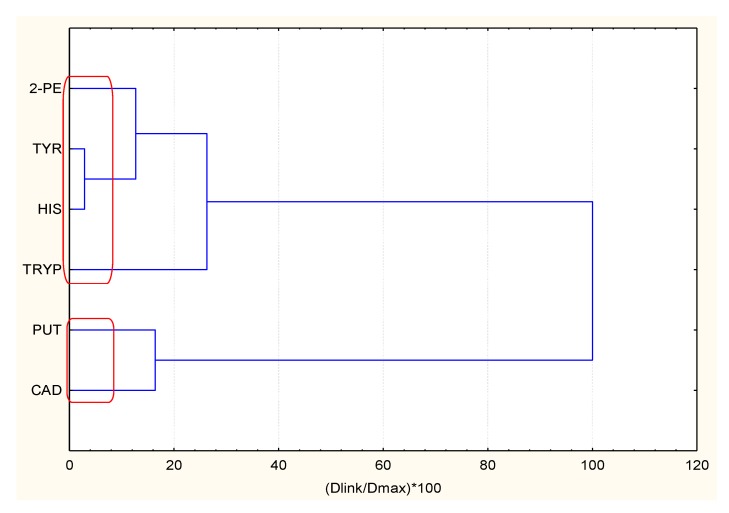
Variable clustering for Wine A.

The example of clustering is presented in Figure 2. The clustering for all wine samples is shown in Supplementary Materials (Figures S1–S5).

It could be concluded that for all wines kept at 4 °C (refrigerator) the data structure is determined by two conditional factors: the one related to the correlation between 2-PE and CAD (“cadaverine factor”) and the other related to the correlation between PUT, HIS, and TRYP (“histamine factor”). TYR is not a significant variable. All these are red wines from REGENT and Frontenac grapes.

For wines kept at room temperature (white wines, SOLARIS and Bianca grapes) the first conditional factor related to wine quality is again “cadaverine factor” but correlated to putrescine; the second is “histamine” factor being correlated strongly to tryptamine. TYR and 2-PE are not significant variables. 

The non-hierarchical clustering confirmed entirely the non-supervised hierarchical procedure. 

#### 2.3.2. Relationship between Production and Storage Conditions for Different Wine Brands

In order to understand the role of the BAs as indicators for wine quality for different conditions of production and storage, the same multivariate statistical analysis was applied to cluster the objects of the study.

**Figure 3:**
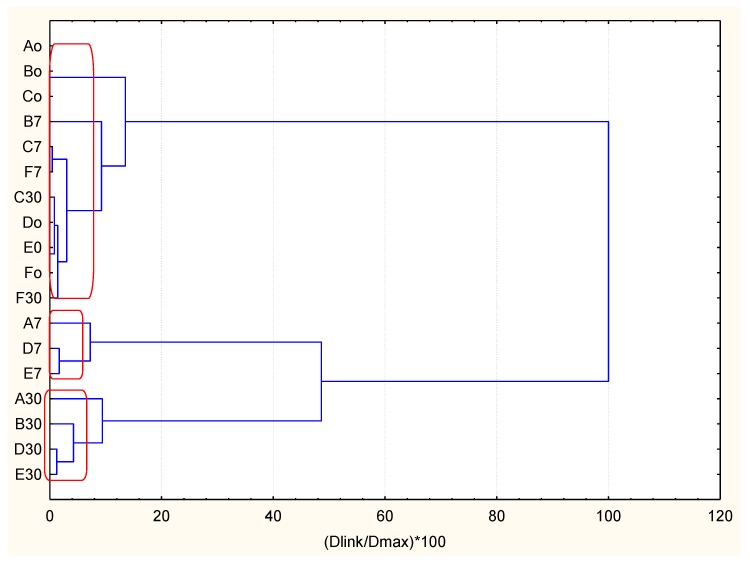
Hierarchical dendrogram for a wine sample (Wine A).

An example of a hierarchical dendrogram for a wine sample (Wine A) is presented in Figure 3. The hierarchical dendrograms for all wine samples is shown in Supplementary Materials (Figures S6–S10).

The results of hierarchical clustering could be summarized as follows (Table 3).

The hierarchical classification is almost the same for each of the brands studied. Cluster 1 includes all samples of bottles opened immediately, the second—after 7 days of storage and the third—after 30 days of storage. It shows convincingly that the role of storage factor is substantial. It is important to note that samples C and F (for 7 and 30 days of storage) belong to Cluster 1 along with the samples after immediate opening. This underlines the significance of the type of stopper as these samples are stoppered by vacuum pump. Once again, the complete similarity of the brands D, E, and F is confirmed. 

**Figure 4:**
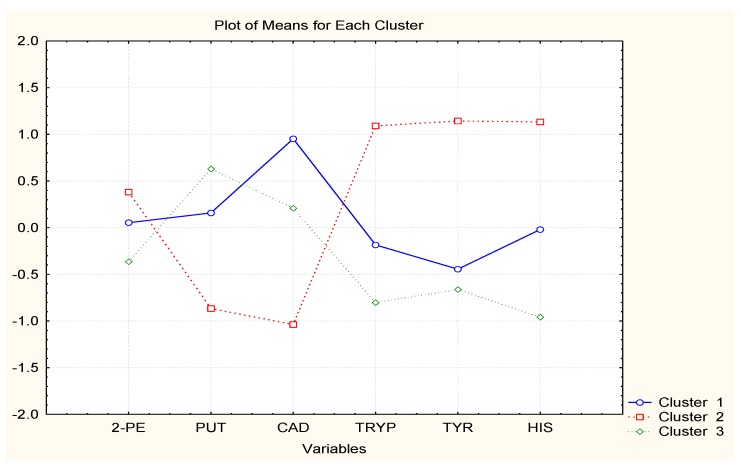
Averages of variables for each cluster (Wine A).

**Figure 5:**
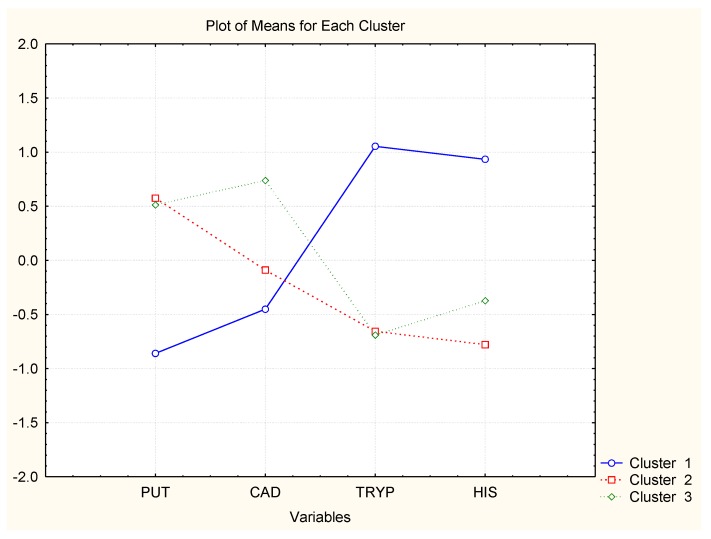
Averages of variables for each cluster (Wine B).

**Figure 6:**
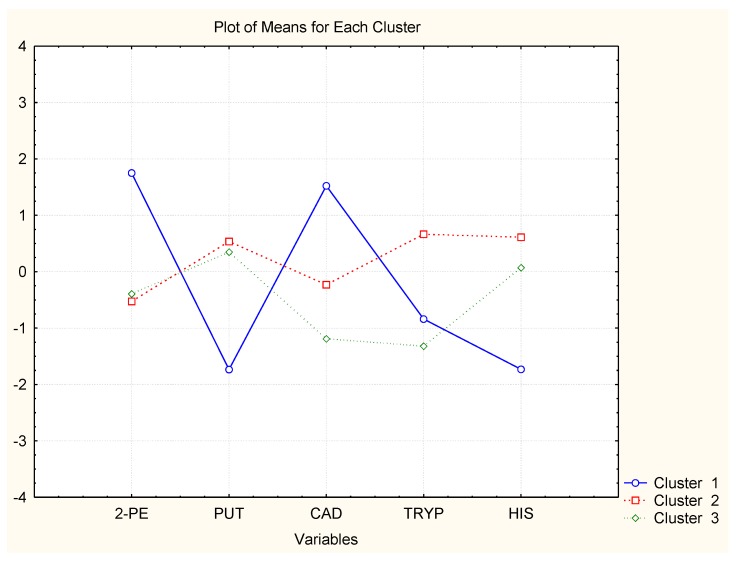
Averages of variables for each cluster (Wines D–F).

In Figure 4, Figure 5 and Figure 6, the averages of each chemical variable for each of the identified clusters of wine samples are shown. The interpretation of the figures aims to reveal if some of the chemical compounds are specifically related to the groups of similarity, i.e., if specific markers could be found among the BAs studied to control the wine quality.

Wine brands D–F have absolutely one and the same classification pattern, cluster 1: pattern 1 (just-opened bottles) indicated by high concentrations of CAD and 2-PE; pattern 2—(7 days of storage after opening) high levels of HIS, TRYP, and PUT; and pattern 3 (30 days of storage) lowest concentrations of all amines. Obviously, the wines lose their amine content after opening. 

The case with the other three wine brands is very similar. Wines B and C have very similar clustering with highest levels of TRYP and HIS for time after opening and lowest PUT and CAD. For the period after opening, it was found that the concentrations of PUT and CAD increase and those of TRYP and HIS decrease. The impact of the other two chemical compounds (2-PE and TYR) is not significant. 

Finally, wine A shows slightly different patterns as all six variables are significant. The just-opened cluster is characterized by highest levels of HIS; the 7 days after opening pattern by highest levels of TRYP, TYR, 2-PE, and HIS, and the last cluster (30 days after opening) by high PUT levels. 

## 3. Materials and Methods 

### 3.1. Reagents and Standards 

Chloroform, pyridine, isobutyl chloroformate (IBCF), and biogenic amine standards (histamine dihydrochloride, cadaverine hydrochloride, putrescine dihydrochloride, tyramine hydrochloride, tryptamine hydrochloride, and 2-phenylethylamine hydrochloride) and internal standard (hexylamine) were obtained from Sigma Aldrich (Steinheim, Germany). Methanol (HPLC grade; purity ≥ 99.8%), 32% hydrochloric acid, and sodium hydroxide (purity 98–100.5%) were obtained from Fluka (St. Louis, MI, USA). Other chemicals were of analytical grade. The solution of alkaline methanol was prepared by dissolving KOH in methanol until saturation. Ultrapure water was obtained from a Milli–Q water purification system (Millipore, Bedford, MA, USA). 

The amine standard solutions (1.0 mg/mL) were prepared individually by dissolving the pure compounds in deionized water. Concentrated solutions of amine standards were prepared by diluting the standard solution with water. The solutions were stored at 4 °C in silanized screw-capped vials with solid PTFE-lined caps (Supelco, Bellefonte, PA, USA).

### 3.2. Samples

A total of 6 samples made from different varieties of grapes were obtained from vineyards in different regions of Poland. The wine samples were considered as follows: non-commercially available white wine comprising with 100% Solaris grapes from West Pomeranian region (Poland), containing 12.9% (*v*/*v*) ethanol and pH 3.09; commercially available white wine comprising with 100% Solaris grapes from Kuyavian-Pomeranian region (Poland), containing 17% (*v*/*v*) ethanol and pH 3.43; commercial white wine comprising with 100% Bianca grapes from Kuyavian-Pomeranian region (Poland), containing 12% (*v*/*v*) ethanol and pH 3.25; commercial red wine elaborated with 100% Regent grapes from Kuyavian-Pomeranian region (Poland), containing 13.5% (*v*/*v*) ethanol and pH 4.02; non-commercial red wine elaborated with 100% Regent grapes from Masovian region (Poland), containing 12% (*v*/*v*) ethanol and pH 3.5; non-commercial red wine comprising with 100% Regent grapes from Masovian region (Poland), containing 12% (*v*/*v*) ethanol and pH 3.5; non-commercial red wine comprising with 100% Frontenac grapes from Masovian region (Poland), containing 13% (*v*/*v*) ethanol and pH 3.37. 

A bottle of each wine was obtained directly from the manufacturer or the owner of the vineyard who produces the wine for his own use in accordance with the practice of wine-making. 

Wine samples were analysed at the moment of opening and thereafter divided into six small bottles and subsequently stoppered. The variables selected for storage conditions were temperature and stopper type. Wine bottles were maintained at room (22 °C) or refrigerated temperature (4 °C), while three strategies were applied to stoppering the bottles: a stopper cork, a stopper screw cap, and a stopper with a vacuum pump that extracts the air from the bottle (Vacu Vin). The samples were coded as A (Room temperature and cork stopper), B (Room temperature and screw cap), C (Room temperature and vacuum), D (refrigeration temperature and cork stopper), E (refrigeration temperature and screw cap), and F (refrigeration temperature and vacuum). 

An aliquot of 50 mL was taken from each bottle 0, 7, and 30 days after it was opened, and immediately frozen. Thirty days were set as the maximum reasonable time for an opened bottle to be consumed since many people store the opened bottles of wine for this extended time. The analysis of BAs from each sample was carried out in duplicate. 

### 3.3. Samples Preparation

The procedure reported by Płotka-Wasylka [17] was applied to determine BAs in wine samples. This procedure is based on dispersive liquid-liquid microextraction (DLLME) for sample preparation and gas chromatography (GC) coupled to mass spectrometry (MS) for final analysis. Five millilitres of sample were placed into a 25 mL screw cap plastic, spiked with IS (50 μL of aqueous solution containing the internal standard at 100 mg/L). A mixture of methanol (dispersive solvent; 215 μL), pyridine:HCl (1:1 *v*/*v*) and IBCF (derivatizing agent; 60 μL) was rapidly injected into the sample tube. After 15 min, 400 μL of chloroform (extractive solvent) was added and shaken by hand (1 min). 150 μL of the bottom layer was taken for further analysis performed by GC-MS. 

### 3.4. GC-MS Analysis

A gas chromatograph 7890A (Agilent Technologies, Santa Clara, CA, USA) equipped with an electronically controlled split/splitless injection port was interfaced to an inert mass selective detector (5975C, Agilent Technologies) with electron impact ionization chamber. GC separation was performed on ZB-5MS capillary column (30 m × 0.25 mm I.D., 0.25 µm film thickness) (Zebron Phenomenex, Shim-Pol, Warsaw, Poland). The injection was made in splitless mode (injection pressure 32 psi) at 230 °C. Helium was the carrier gas with a constant pressure of 30 psi. The oven temperature program was as follows: 50 °C held for 1min, ramped to 280 °C at 15 °C/min and held for 9 min (280 °C). Total run time was 25.3 min. The MS transfer line temperature was held at 280 °C. Mass spectrometric parameters were set as follows: electron impact ionization with 70 eV energy; ion source temperature, 250 °C. The MS system was routinely set in selective ion monitoring mode and each analyte was quantified based on peak area using one target and one or more qualifier ion(s) (Table 4). All the ion fragments with their relative intensities at the specific retention times were considered as valid confirmation criterion and used for the identification of the specific BAs. An Agilent ChemStation was used for data collection and GC-MS control. 

### 3.5. Quality Assurance

The method linearity was determined by a regression analysis of the relative area (ratio between peak area of BAs to the peak area of the IS) versus the amine concentration. Thus, ten aqueous solutions containing all analytes with concentrations ranging from 0.05 to 1.0 mg/L and 1.0 to 10.0 mg/L were submitted to the whole analytical procedure. The results obtained showed that linearity was excellent for all the compounds with correlation coefficients (R^2^) ranging from 0.9968 to 0.9989. The recovery was determined by comparing unspiked wine samples to spiked for two concentration levels (0.05 mg/L and 0.25 mg/L; n = 4). The average recovery values ranged from 76% to 99% as can be seen in Table 2. The intra-day precision was determined by analysing in the same day four replicates of wine samples spiked at two levels (0.05 mg/L and 0.25 mg/L); each replicate was submitted to the overall developed method. Inter-day precision was determined by analysis of samples on two different days over a period of three weeks. The relative standard deviation (RSD) for inter-day precision ranged from 5% to 10% and for intra-day precision ranged from 4% to 12% (Table 5). The limits of detection (LOD) of the developed method were determined by analyses of chromatographic extracts of aqueous solution spiked with decreasing amounts of the analytes until a signal-to-noise ratio 3:1 was reached. The limits of quantification (LOQs) were determined considering a signal-to-noise ratio of 10:1. The LODs ranged from 1.4 to 4.2 µg/L and the LOQs ranged from 4.6 to 12.6 µg/L. 

### 3.6. Chemometric Analysis

Cluster analysis (hierarchical and non-hierarchical clustering) is one of the most frequently applied chemometric methods for multivariate data interpretation [19]. Hierarchical cluster analysis is thoroughly described as an unsupervised pattern recognition approach since non-hierarchical clustering is a typical supervised method. Both approaches make it possible to reveal groups of similarity (clusters) within a large and, generally, diffuse data set. The cluster formation could be achieved with respect to the objects of interest (described by various parameters, features, variables) or with respect to the variables identifying the objects. In order to perform the hierarchical clustering procedure several steps are necessary—data standardization (in order to eliminate the role of variable dimension on the clustering), determination of the distances between the objects by some similarity measure equation (usually Euclidean distances), and linkage of similar (close) objects in clusters (often the Ward’s method is preferred). The graphical output of the analysis is a tree-like diagram called a dendrogram. Usually, statistical significance of the clusters has to be determined in order to better identify significant clusters. In the non-hierarchical clustering approach, the members of the pre-defined clusters are automatically given, as well as the average values of the variables for each cluster. Missing data are replaced by the value LOD/2. This substitution is already obligatory for replacing missing data. If zero is introduces the software automatically deletes the whole object and there is loss of information. Any other value (small one) is possible but LOD/2 is logical, especially in cases where the analyte is probably not detected due to instrumental problems. The software package used was STATISTICA 8.0 (TIBCO Software Inc., Palo Alto, CA, USA).

## 4. Conclusions

It is common for consumers to drink wine several days after opening the bottle and having sometimes stored it at room temperature. Moreover, in the restaurant sector, wine is also often kept in opened bottles, thus it is important to monitor the levels of BAs in wine stored in opened bottles under different conditions. This work evaluates the profile of selected BAs in opened bottles of wine kept in different storage conditions (six samples). From the data obtained in this study, the following conclusions could be reached:

- slight changes were observed in the profiles of BAs and pH;
- the type of stopper impacted the concentration of all BAs;
- all red wines displayed the same trend in changes of concentration of appropriate BAs;
- white wine showed a clear and significant trend in the total content of BAs, however, this trend differed depending on the type of BAs;
- the concentration of only one compound, namely putrescine, decreased in all wine samples over time, no matter if it was red or white wine;
- chemometric analysis confirmed that the samples were grouped according to their storage time and the storage conditions.

Generally, total concentrations of BAs in red wines are higher than in white wines. This fact is due to the malolactic fermentation that takes place in all red wines and just in some white wines, converting malic acid into lactic acid and increasing the medium pH. This reaction is performed by lactic acid bacteria, which are the main producers of BAs and, in addition, their decarboxylase enzymes improve their activity with a higher pH. In addition, the obtained results suggest that the concentrations of BAs in opened wine bottles suffered slight changes during storage. 

Further analysis of chemical stability together with microbiology research is therefore recommended to determine which factors significantly affect the evolution of BAs during storage. In addition, it is recommended to analyse more samples to confirm the results obtained here.

## Figures and Tables

**Table 1:** Evolution of BA concentrations and pH in standard and high quality red wines and young white wine in different storage conditions.

A	Non-Commercially Available Wine Made from 100% SOLARIS Grapes (Mean Concentration (µg/L) ± Standard Deviation)																	
Analytes	A0	A7	A30	B0	B7	B30	C0	C7	C30	D0	D7	D30	E0	E7	E30	F0	F7	F30
2-PE	43.02 ± 0.21 ^a^	51.11 ± 0.32 ^a,b^	40.16 ± 0.19 ^b^	43.02 ± 0.21 ^a^	47.45 ± 0.34 ^a,b^	42.23 ± 0.45 ^b^	43.02 ± 0.21	44.09 ± 0.38	43.96 ± 0.45	43.17 ± 0.32 ^a^	48.11 ± 0.42 ^b^	42.12 ± 0.38 ^b^	43.17 ± 0.32	46.32 ± 0.23 ^b^	42.34 ± 0.28 ^b^	43.17 ± 0.32	43.12 ± 0.28	42.99 ± 0.43
PUT	62.12 ± 0.78 ^c^	60.32 ± 0.27 ^b^	55.87 ± 0.25 ^b,c^	62.12 ± 0.78 ^c^	60.76 ± 0.65	58.65 ± 0.56 ^c^	62.12 ± 0.78	61.00 ± 0.48	60.09 ± 0.44	60.72 ± 0.73 ^c^	59.32 ± 0.67	57.43 ± 0.54 ^c^	60.72 ± 0.73	60.32 ± 0.58	58.43 ± 0.48	60.72 ± 0.73	61.09 ± 0.37	59.19 ± 0.35
CAD	32.08 ± 0.45	31.36 ± 0.32	31.08 ± 0.28	32.08 ± 0.45	33.09 ± 0.54	32.23 ± 0.46	32.08 ± 0.45	33.31 ± 0.57	32.87 ± 0.48	32.76 ± 0.49	32.11 ± 0.39	31.67 ± 0.43	32.76 ± 0.49	31.98 ± 0.32	31.87 ± 0.31	32.76 ± 0.49	32.99 ± 0.45	32.57 ± 0.32
TRYP	133.0 ± 1.6 ^a,c^	156.0 ± 2.1 ^a,b^	178.4 ± 2.5 ^b,c^	133.0 ± 1.6 ^a,c^	143.7 ± 1.5 ^b^	157.09 ± 1.7 ^b,c^	133.0 ± 1.6	136.3 ± 1.6	137.0 ± 2.1	132.8 ± 1.4 ^a,c^	152.7 ± 1.8 ^b^	166.8 ± 2.1 ^b,c^	132.8 ± 1.4 ^a,c^	147.2 ± 2.1 ^a,b^	155.3 ± 2.0 ^b,c^	132.8 ± 1.4	135.3 ± 2.1	136.4 ± 2.4
TYR	24.01 ± 0.18 ^a^	35.65 ± 0.11 ^a,b^	27.43 ± 0.12 ^b^	24.01 ± 0.18	27.07 ± 0.23	23.09 ± 0.17	24.01 ± 0.18	25.09 ± 0.16	24.89 ± 0.17	24.32 ± 0.21 ^a^	33.45 ± 0.16 ^a,b^	27.98 ± 0.14 ^b^	24.32 ± 0.21 ^a,c^	31.09 ± 0.17 ^a^	28.21 ± 0.19 ^c^	24.32 ± 0.21	24.78 ± 0.12	24.49 ± 0.12
HIS	416 ± 13 ^a,,c^	552 ± 15 ^a,b^	482 ± 13 ^b,c^	416 ± 13 ^a,c^	489 ± 20 ^a^	463 ± 18 ^c^	416 ± 13 ^a,c^	452 ± 17 ^a,b^	438 ± 20 ^b,c^	421 ± 20 ^a,c^	523 ± 17 ^a,b^	496 ± 19 ^b,c^	421 ± 20 ^a,c^	503 ± 21 ^a,b^	484 ± 20 ^b,c^	421 ± 20 ^a^	448 ± 27 ^a,b^	418 ± 21 ^b^
TOTAL	710 ^a,c^	886 ^a,b^	815 ^b,c^	710 ^a,c^	801 ^a^	776 ^c^	710 ^a,c^	752 ^a,b^	737 ^b,c^	715 ^a,c^	849 ^a,b^	822 ^b,c^	715 ^a,c^	820 ^a^	800 ^c^	715 ^a^	745 ^a,b^	713 ^b^
pH	3.09 ± 0.01	3.07 ± 0.01	3.06 ± 0.01	3.09 ± 0.01	3.09 ± 0.01	3.08 ± 0.01	3.09 ± 0.01	3.07 ± 0.01	3.08 ± 0.01	3.09 ± 0.01	3.07 ± 0.01	3.09 ± 0.01	3.09 ± 0.01	3.07 ± 0.01	3.07 ± 0.01	3.09 ± 0.01	3.07 ± 0.01	3.08 ± 0.01
B	Commercially Available Wine Made from 100% SOLARIS Grapes (Mean Concentration (µg/L) ± Standard Deviation)																	
2-PE	<LOD	<LOD	<LOD	<LOD	<LOD	<LOD	<LOD	<LOD	<LOD	<LOD	<LOD	<LOD	<LOD	<LOD	<LOD	<LOD	<LOD	<LOD
PUT	759 ± 21 ^a,c^	700 ± 26 ^a,b^	612 ± 23 ^b,c^	759 ± 21 ^a,c^	711 ± 24 ^a,b^	634 ± 21^b,c^	759 ± 21 ^c^	745 ± 23	730 ± 19 ^c^	756 ± 23 ^a,c^	738 ± 24 ^a,b^	650 ± 20 ^b,c^	756 ± 23 ^c^	746 ± 23 ^b^	700 ± 26 ^b,c^	756 ± 23	751 ± 20	748 ± 23
CAD	12.00 ± 0.12	11.89 ± 0.09	11.91 ± 0.11	12.00 ± 0.12	12.13 ± 0.14	12.01 ± 0.11	12.00 ± 0.12	11.87 ± 0.14	11.85 ± 0.09	11.80 ± 0.14	11.76 ± 0.11	11.00 ± 0.13	11.80 ± 0.14	11.78 ± 0.13	11.69 ± 0.16	11.80 ± 0.14	11.79 ± 0.11	11.79 ± 0.13
TRYP	30.15 ± 0.17 ^a,c^	51.21 ± 0.21^a,b^	74.32 ± 0.23 ^b,c^	30.15 ± 0.17 ^a,c^	40.09 ± 0.23 ^a,c^	54.12 ± 0.25 ^b,c^	30.15 ± 0.17 ^c^	33.78 ± 0.18	35.01 ± 0.18 ^c^	31.05 ± 0.15 ^a,c^	47.84 ± 0.65 ^a,b^	65.09 ± 0.56 ^b,c^	31.05 ± 0.15 ^a,c^	45.67 ± 0.15 ^a,b^	52.08 ± 0.23 ^b,c^	31.05 ± 0.15 ^a,c^	35.67 ± 0.18 ^a^	36.10 ± 0.13 ^c^
TYR	<LOD	<LOD	<LOD	<LOD	<LOD	<LOD	<LOD	<LOD	<LOD	<LOD	<LOD	<LOD	<LOD	<LOD	<LOD	<LOD	<LOD	<LOD
HIS	128.0 ± 2.0 ^a,c^	234.26 ± 4.1 ^a,b^	163.43 ± 4.1 ^b,c^	128.0 ± 4.0 ^a,c^	189.98 ± 4.4 ^b,c^	160.54 ± 3.9 ^b,c^	128.0 ± 4.0 ^a,c^	169.0 ± 3.9 ^a,b^	146.8 ± 4.0 ^b,c^	127.7 ± 4.1 ^a,c^	227.1 ± 4.3 ^a,b^	201.0 ± 3.8^b,c^	127.7 ± 4.1 ^a,c^	201.0 ± 5.0 ^a,b^	185.5 ± 4.6 ^b,c^	127.7 ± 4.1 ^a^	143.9 ± 3.7 ^a,b^	128.7 ± 3.9 ^b^
TOTAL	929 ^a,c^	997 ^a,b^	861.66 ^b,c^	929 ^c^	953 ^b^	860.67 ^b,c^	929 ^a^	960 ^a,b^	923.67 ^b^	927 ^a^	1025 ^a,b^	927^b^	927 ^a,c^	1004 ^a,b^	949 ^b,c^	927 ^a^	942 ^a,b^	925 ^b^
pH	3.43 ± 0.01	3.41 ± 0.01	3.42 ± 0.01	3.43 ± 0.01	3.40 ± 0.01	3.41 ± 0.01	3.43 ± 0.01	3.42 ± 0.01	3.40 ± 0.01	3.39 ± 0.01	3.42 ± 0.01	3.43 ± 0.01	3.41 ± 0.01	3.40 ± 0.01	3.39 ± 0.01	3.42 ± 0.01	3.40 ± 0.01	3.41 ± 0.01
C	Commercially Available Wine Made from 100% BIANCA Grapes (Mean Concentration (µg/L) ± Standard Deviation)																	
2-PE	<LOD	<LOD	<LOD	<LOD	<LOD	<LOD	<LOD	<LOD	<LOD	<LOD	<LOD	<LOD	<LOD	<LOD	<LOD	<LOD	<LOD	<LOD
PUT	260 ± 10 ^a,c^	201 ± 13 ^a,b^	131 ± 16 ^b,c^	260 ± 10 ^a,c^	221 ± 11 ^a,b^	157 ± 13 ^b,c^	260 ± 10 ^a,c^	245 ± 11 ^a,b^	228 ± 16 ^b,c^	259 ± 11 ^a,c^	237 ± 14 ^a,b^	156 ± 11 ^b,c^	259 ± 11 ^a,c^	231 ± 13 ^a,b^	178 ± 10 ^b,c^	259 ± 11 ^c^	250 ± 14	245 ± 10 ^c^
CAD	<LOD	<LOD	<LOD	<LOD	<LOD	<LOD	<LOD	<LOD	<LOD	<LOD	<LOD	<LOD	<LOD	<LOD	<LOD	<LOD	<LOD	<LOD
TRYP	10.11 ± 0.10 ^a,c^	23.01 ± 0.11 ^a,b^	37.91 ± 0.17 ^b,c^	10.11 ± 0.10 ^a,c^	21.00 ± 0.14 ^a,b^	35.13 ± 0.14 ^b,c^	10.11 ± 0.10 ^a,c^	13.45 ± 0.13 ^a^	15.14 ± 0.11 ^c^	10.14 ± 0.11 ^a,c^	22.00 ± 0.14 ^a,b^	35.09 ± 0.11 ^b,c^	10.14 ± 0.11 ^a,c^	19.76 ± 0.16 ^b,c^	33.12 ± 0.14 ^b,c^	10.14 ± 0.11 ^c^	11.99 ± 0.11	13.56 ± 0.10 ^c^
TYR	<LOD	<LOD	<LOD	<LOD	<LOD	<LOD	<LOD	<LOD	<LOD	<LOD	<LOD	<LOD	<LOD	<LOD	<LOD	<LOD	<LOD	<LOD
HIS	172.1 ± 3.2 ^a,c^	258.1 ± 4.0 ^a,b^	197.7 ± 3.9 ^b,c^	172.1 ± 3.2 ^a,c^	241.9 ± 4.5 ^a,b^	210.1 ± 4.1 ^b,c^	172.1 ± 3.2 ^a,c^	209.0 ± 3.7 ^a,b^	189.1 ± 4.1 ^b,c^	171.9 ± 3.3 ^a,c^	240.5 ± 4.2 ^a,b^	210.4 ± 3.5 ^b,c^	171.9 ± 3.3 ^a,c^	227.8 ± 4.0 ^a,b^	214.7 ± 4.6 ^b,c^	171.9 ± 3.3 ^a^	189.0 ± 4.7 ^a,b^	176.9 ± 3.7 ^b^
TOTAL	442 ^a,c^	482 ^a,b^	367 ^b,c^	442 ^a,c^	484 ^a,b^	402^b,c^	442 ^a^	467 ^a,b^	432 ^b^	441 ^a,c^	499 ^a,b^	402 ^b,c^	441 ^a^	479 ^a,b^	426^b^	441	451	435
pH	3.25 ± 0.01	3.24 ± 0.01	3.26 ± 0.01	3.25 ± 0.01	3.22 ± 0.01	3.24 ± 0.01	3.25 ± 0.01	3.24 ± 0.01	3.23 ± 0.01	3.25 ± 0.01	3.24 ± 0.01	3.26 ± 0.01	3.25 ± 0.01	3.23 ± 0.01	3.24 ± 0.01	3.25 ± 0.01	3.24 ± 0.01	3.26 ± 0.01
D	Commercially Available Wine Made from 100% REGENT Grapes (Mean Concentration (µg/L) ± Standard Deviation)																	
2-PE	19.23 ± 0.16 ^c^	20.15 ± 0.18 ^b^	30.12 ± 0.21 ^b,c^	19.23 ± 0.16 ^c^	20.09 ± 0.18 ^b^	28.01 ± 0.20 ^b,c^	19.23 ± 0.16 ^c^	19.78 ± 0.15 ^b^	23.09 ± 0.17 ^b,c^	19.43 ± 0.19 ^c^	20.01 ± 0.21 ^b^	28.31 ± 0.21 ^b,c^	19.43 ± 0.19 ^c^	19.91 ± 0.22 ^b^	26.09 ± 0.18 ^b,c^	19.43 ± 0.19	19.56 ± 0.20	21.19 ± 0.22
PUT	298.2 ± 6.8 ^c^	291.1 ± 7.0 ^b^	211.9 ± 6.8 ^b,c^	298.2 ± 6.8 ^c^	293.6 ± 7.1 ^b^	230.3 ± 6.7 ^b,c^	298.2 ± 6.8	296.2 ± 3.0	286.8 ± 3.7	297.3 ± 7.2 ^c^	293.1 ± 8.0 ^b^	234.81 ± 7.6 ^b,c^	297.3 ± 7.2 ^c^	295.2 ± 8.1 ^b^	254.2 ± 7.1 ^b,c^	297.3 ± 7.2 ^c^	296.5 ± 7.1 ^b^	290.4 ± 8.1 ^b,c^
CAD	35.89 ± 0.43 ^a,c^	30.81 ± 0.38 ^a,b^	45.87 ± 0.41 ^b,c^	35.89 ± 0.43 ^a,c^	32.22 ± 0.35 ^a,b^	38.45 ± 0.37 ^b,c^	35.89 ± 0.43	34.12 ± 0.36	36.09 ± 0.38	36.01 ± 0.51 ^a,b^	32.89 ± 0.60 ^a,b^	42.89 ± 0.49 ^b,c^	36.01 ± 0.51 ^a^	32.90 ± 0.54 ^b^	37.12 ± 0.60 ^b,c^	36.01 ± 0.51	35.15 ± 0.52	36.12 ± 0.54
TRYP	4.32 ± 0.11 ^a,c^	2.32 ± 0.12 ^a^	2.30 ± 0.16^c^	4.32 ± 0.11 ^a,c^	2.78 ± 0.17 ^a^	2.89 ± 0.18 ^c^	4.32 ± 0.11	4.27 ± 0.12	4.22 ± 0.10	4.22 ± 0.15 ^a,c^	2.78 ± 0.17	2.80 ± 0.16^c^	4.22 ± 0.15 ^a,c^	3.11 ± 0.18 ^a^	3.09 ± 0.19 ^c^	4.22 ± 0.15	4.12 ± 0.17	4.09 ± 0.19
TYR	<LOD	<LOD	<LOD	<LOD	<LOD	<LOD	<LOD	<LOD	<LOD	<LOD	<LOD	<LOD	<LOD	<LOD	<LOD	<LOD	<LOD	<LOD
HIS	311.3 ± 7.7 ^c^	300 ± 8.1 ^b^	250.2 ± 7.9 ^b,c^	311.3 ± 7.7 ^c^	306.0 ± 6.5 ^b^	276.0 ± 7.1 ^b,c^	311.3 ± 6.7	310.3 ± 8.1	308.9 ± 7.9	309.3 ± 6.9 ^c^	301.1 ± 6.5 ^b^	265.9 ± 8.1 ^b,c^	309.3 ± 6.9 ^c^	305.5 ± 8.5 ^b^	280.1 ± 7.8 ^b,c^	309.3 ± 7.9	308.1 ± 8.1	306.1 ± 7.9
TOTAL	669 ^a,c^	644 ^a,b^	540 ^b,c^	669	655 ^b^	576 ^b,c^	669	664	659	666 ^a,c^	650 ^a,b^	575 ^b,c^	666 ^c^	657 ^b^	602 ^b,c^	666	663	657
pH	4.02 ± 0.01	4.06 ± 0.01	4.09 ± 0.01	4.02 ± 0.01	4.07 ± 0.01	4.10 ± 0.01	4.02 ± 0.01	4.06 ± 0.01	4.09 ± 0.01	3.99 ± 0.01	3.97 ± 0.01	3.94 ± 0.01	3.99 ± 0.01	3.96 ± 0.01	3.94 ± 0.01	3.99 ± 0.01	3.97 ± 0.01	3.95 ± 0.01
E	Non-Commercially Available Wine Made from 100% REGENT Grapes (Mean Concentration (µg/L) ± Standard Deviation)																	
2-PE	21.17 ± 0.20 ^c^	22.18 ± 0.22 ^b^	29.09 ± 0.19 ^b,c^	21.17 ± 0.20 ^c^	21.98 ± 0.22 ^b^	27.78 ± 0.19 ^b,c^	21.17 ± 0.20 ^c^	21.56 ± 0.21	22.87 ± 0.19 ^c^	21.43 ± 0.21 ^a,c^	22.34 ± 0.23 ^a,b^	30.09 ± 0.19 ^b,c^	21.43 ± 0.21 ^c^	21.9 ± 0.23 ^b^	27.67 ± 0.27 ^b,c^	21.43 ± 0.21 ^c^	21.56 ± 0.19 ^b^	22.09 ± 0.24 ^b,c^
PUT	289.9 ± 8.5 ^a,c^	280.98 ± 9.1 ^a,b^	202.78 ± 6.8 ^b,c^	289.9 ± 8.5 ^c^	282.78 ± 8.1^b^	221.9 ± 7.9 ^b,c^	289.9 ± 8.5 ^c^	286.2 ± 8.1	278.2 ± 8.5 ^c^	285.4 ± 7.7 ^c^	281.09 ± 8.0 ^b^	220.19 ± 6.9 ^b,c^	285.4 ± 7.7 ^c^	283.12 ± 8.1 ^b^	245.23 ± 7.8 ^b,c^	285.4 ± 7.7	284.1 ± 7.8	279.09 ± 8.1
CAD	31.09 ± 0.21 ^a,c^	26.16 ± 0.23 ^a,b^	41.78 ± 0.19 ^b,c^	31.09 ± 0.21 ^a,c^	28.12 ± 0.25 ^a,b^	36.14 ± 0.21 ^b,c^	31.09 ± 0.21	29.97 ± 0.20	31.76 ± 0.19	31.16 ± 0.24 ^a,c^	27.98 ± 0.19 ^a,b^	37.78 ± 0.30 ^b,c^	31.16 ± 0.24 ^a,c^	28.34 ± 0.19 ^a,b^	33.45 ± 0.21 ^b,c^	31.16 ± 0.24^a^	30.01 ± 0.27 ^a,b^	31.46 ± 0.22 ^b^
TRYP	3.21 ± 0.11 ^a,c^	2.01 ± 0.10 ^a^	2.10 ± 0.11 ^c^	3.21 ± 0.11 ^a,c^	2.45 ± 0.13 ^a^	2.44 ± 0.12 ^c^	3.21 ± 0.11	3.15 ± 0.10	3.12 ± 0.11	3.11 ± 0.17 ^a,c^	1.70 ± 0.16 ^a^	1.73 ± 0.17 ^c^	3.11 ± 0.17 ^a,c^	2.11 ± 0.16 ^a^	2.09 ± 0.16 ^c^	3.11 ± 0.17	3.02 ± 0.18	2.98 ± 0.15
TYR	<LOD	<LOD	<LOD	<LOD	<LOD	<LOD	<LOD	<LOD	<LOD	<LOD	<LOD	<LOD	<LOD	<LOD	<LOD	<LOD	<LOD	<LOD
HIS	326.0 ± 6.9 ^c^	315.0 ± 6.6 ^b^	265.1 ± 7.9 ^b,c^	326.0 ± 9.0 ^c^	321.8 ± 6.3 ^b^	291.1 ± 7.1 ^b,c^	326.0 ± 6.9	325.3 ± 7.4	322.0 ± 7.1	324.9 ± 7.4 ^a,c^	315.01 ± 6.9 ^a,b^	280.9 ± 7.9 ^b,c^	324.9 ± 7.4 ^c^	319.0 ± 7.1 ^b^	295.1 ± 6.8 ^b,c^	324.9 ± 7.4	323.78 ± 8.1	321.1 ± 8.0
TOTAL	671 ^a,c^	646 ^a,b^	541 ^b,c^	671 ^a,c^	657 ^a,b^	579 ^b,c^	671	666	657	666 ^a,c^	648 ^a,b^	571 ^b,c^	666 ^c^	654 ^b^	604 ^b,c^	666	662	657
pH	3.50 ± 0.01	3.52 ± 0.01	3.57 ± 0.01	3.50 ± 0.01	3.53 ± 0.01	3.57 ± 0.01	3.50 ± 0.01	3.52 ± 0.01	3.56 ± 0.01	3.49 ± 0.01	3.48 ± 0.01	3.46 ± 0.01	3.49 ± 0.01	3.47 ± 0.01	3.45 ± 0.01	3.49 ± 0.01	3.46 ± 0.01	3.44 ± 0.01
F	Non-Commercially Available Wine Made from 100% FRONTENAC Grapes (Mean Concentration (µg/L) ± Standard Deviation)																	
2-PE	24.31 ± 0.22 ^c^	25.46 ± 0.23 ^b^	30.23 ± 0.19 ^b,c^	24.31 ± 0.22 ^c^	24.98 ± 0.22 ^b^	30.17 ± 0.27 ^b,c^	24.31 ± 0.22 ^c^	24.67 ± 0.31 ^b^	26.01 ± 0.27 ^b,c^	24.17 ± 0.27 ^c^	24.15 ± 0.32 ^b^	29.56 ± 0.26 ^b,c^	24.17 ± 0.27 ^c^	24.76 ± 0.24 ^b^	28.43 ± 0.26 ^b,c^	24.17 ± 0.27 ^c^	24.35 ± 0.19 ^b^	25.00 ± 0.21 ^b,c^
PUT	482 ± 13 ^c^	471 ± 16 ^b^	389 ± 14 ^b,c^	482 ± 13 ^c^	474 ± 12 ^b^	416 ± 14 ^b,c^	482 ± 13	479 ± 18	471 ± 15	481 ± 11 ^c^	477 ± 13 ^b^	416 ± 15 ^b,c^	481 ± 11 ^c^	479 ± 12 ^b^	435 ± 11 ^b,c^	481 ± 11	480 ± 14	476 ± 11
CAD	96.01 ± 0.91 ^a,c^	90.2 ± 1.2 ^a,b^	107.2 ± 1.4 ^b,c^	96.01 ± 0.91 ^c^	94.7 ± 1.0 ^b^	102.1 ± 2.0 ^b,c^	96.01 ± 0.91	95.7 ± 1.4	96.7 ± 1.2	95.87 ± 0.78 ^c^	91.80 ± 0.56 ^b^	101.1 ± 1.1 ^b,c^	95.87 ± 0.78 ^c^	95.09 ± 12 ± 0.98 ^b^	100.0 ± 1.1 ^b,c^	95.87 ± 0.78	95.82 ± 0.98	95.97 ± 0.95
TRYP	3.04 ± 0.10 ^a,c^	2.10 ± 0.11 ^a^	2.19 ± 0.13 ^c^	3.04 ± 0.10 ^a,c^	2.56 ± 0.13 ^a^	2.54 ± 0.11 ^c^	3.04 ± 0.10	2.98 ± 0.11	2.96 ± 0.12	3.00 ± 0.13 ^a,c^	2.45 ± 0.13 ^a^	2.53 ± 0.10 ^c^	3.00 ± 0.13 ^a,c^	2.74 ± 0.16 ^a^	2.81 ± 0.11 ^c^	3.00 ± 0.13	2.99 ± 0.11	2.96 ± 15
TYR	<LOD	<LOD	<LOD	<LOD	<LOD	<LOD	<LOD	<LOD	<LOD	<LOD	<LOD	<LOD	<LOD	<LOD	<LOD	<LOD	<LOD	<LOD
HIS	1639 ± 48 ^a,c^	1578 ± 51 ^a,b^	1415 ± 47 ^b,c^	1639 ± 48 ^c^	1602 ± 45 ^b^	1454 ± 43 ^b,c^	1639 ± 48	1625 ± 48	1613 ± 51	1637 ± 51 ^a,c^	1592 ± 48 ^a,b^	1465 ± 51 ^b,c^	1637 ± 51 ^c^	1612 ± 47 ^b^	1498 ± 50 ^b,c^	1637 ± 51	1631 ± 47	1625 ± 42
TOTAL	2244 ^a,c^	2167 ^a,b^	1944 ^b,c^	2244 ^c^	2198 ^b^	2005 ^b,c^	2244	2227	2210	2241 ^a,c^	2187 ^a,b^	2014 ^b,c^	2241 ^c^	2214 ^b^	2064 ^b,c^	2241	2234	2225
pH	3.37 ± 0.01	3.38 ± 0.01	3.40 ± 0.01	3.37 ± 0.01	3.37 ± 0.01	3.39 ± 0.01	3.37 ± 0.01	3.38 ± 0.01	3.40 ± 0.01	3.36 ± 0.01	3.35 ± 0.01	3.34 ± 0.01	3.36 ± 0.01	3.34 ± 0.01	3.33 ± 0.01	3.36 ± 0.01	3.35 ± 0.01	3.34 ± 0.01

Superscript “a” shows significant differences between day 0 and day 7 according to ANOVA test (*p* < 0.05). Superscript “b” shows significant differences between day 7 and day 30 according to ANOVA test (*p* < 0.05). Superscript “c” shows significant differences between day 0 and day 30 according to ANOVA test (*p* < 0.05).

**Table 2:** Cluster composition for variables for 6 wine brands.

Brand	Cluster 1 Cadaverine Factor	Cluster 2 Histamine Factor
Wine A	PUT CAD	2-PE TYR HIS TRYP
Wine B	PUT CAD	TRYP HIS
Wine C	PUT	TRYP HIS
Wine D	2-PE CAD	PUT HIS TRYP
Wine E	2-PE CAD	PUT HIS TRYP
Wine F	2-PE CAD	PUT HIS TRYP

**Table 3:** Cluster content for all wine brands.

Brand	Cluster 1	Cluster 2	Cluster 3
Wine A	A0, B0, C0, D0, E0, F0, B7, C7, F7, C30, F30	A7, D7, E7	A30,B30, D30, E30
Wine B	A0, B0, C0, D0, E0, F0, C7, F7, C30, F30	A7, B7, D7, E7, E30	A30,B30, D30, E30
Wine C	A0, B0, C0, D0, E0, F0, C7, F7, C30, F30	A7, B7, D7, E7	A30,B30, D30, E30
Wine D	A0, B0, C0, D0, E0, F0, C7, F7, C30, F30	A7, B7, D7, E7	A30,B30, D30, E30
Wine E	A0, B0, C0, D0, E0, F0, C7, F7, C30, F30	A7, B7, D7, E7	A30,B30, D30, E30
Wine F	A0, B0, C0, D0, E0, F0, C7, F7, C30, F30	A7, B7, D7, E7	A30,B30, D30, E30

**Table 4:** Fragments, relative intensities, and retention time (t_R_) of BAs obtained by application of GC-MS technique.

Analytes	*m*/*z* SIM Ions					t_R_ [min]
Hexylamine	146 (99.9)	130 (76.7)	128 (14.8)			7.893
2-phenylethylamine	130(99.9)	104(79.6)	91 (76.4)	221 (30.7)	148 (18.5)	10.016
Putrescine	170 (99.9)	130 (63.6)	288 (12)			11.773
Tryptamine	130 (99.9)	143 (59.2)	260 (19.1)	187 (4.1)		13.212
Tyramine	120 (99.9)	107 (27.7)	176 (4.6)	237 (2.2)	337 (1.4)	13.319
Cadaverine	130 (79)	84 (82)	129 (73)	302 (2)		13.505
Histamine	194 (99.9)	238 (16.7)	138 (25.8)			14.168

Note: *m*/*z* SIM ions represented in bold were used as quantitative ions, while the other were used as qualitative ions.

**Table 5:** Information on average recoveries (%), intra-day repeatability (%RSD), inter-day repeatability (%RSD), and limits of detection (LOD, (µg/L) and limits of quantification (LOQ, **(**µg/L)) obtained with the optimized method in spiked wine samples, analysed by GC-MS (n = 4 at each level).

Analyte	Concentration Levels				Interday (%RSD)	LOD (µg/L)	LOQ (µg/L)
	0.05 mg/L		0.25 mg/L				
	Recovery (%)	Intraday (%RSD)	Recovery (%)	Intraday (%RSD)			
CAD	83	6	92	7	6	1.5	4.5
HIST	76	5	88	5	7	4.2	12.6
PUT	98	8	103	7	8	1.4	4.6
TRP	83	12	89	8	10	1.6	4.8
TYR	99	5	105	4	5	3.3	9.9
2-PE	88	6	97	6	6	3.2	9.6

## Supplementary Materials

The following are available online, Figure S1: Variable clustering for Wine B, Figure S2: Variable clustering for Wine C, Figure S3: Variable clustering for Wine D, Figure S4: Variable clustering for Wine E, Figure S5: Variable clustering for Wine F, Figure S6: Hierarchical dendrogram for wine samples (Wine B), Figure S7: Hierarchical dendrogram for wine samples (Wine C), Figure S8: Hierarchical dendrogram for wine samples (Wine D), Figure S9: Hierarchical dendrogram for wine samples (Wine E), Figure S10: Hierarchical dendrogram for wine samples (Wine F).

## References

[1] Stadnik J., Dolatowski Z.J. (2010). Biogenic amines in meat and fermented meat products. Acta Sci. Pol. Technol. Aliment..

[2] Peña-Gallego A., Hernandez-Orte P., Cacho J., Ferreira V. (2012). High-performance liquid chromatography analysis of amines in must and wine: A review. Food Rev. Int..

[3] Köse S., Witczak A., Sikorski Z. (2017). Biogenic Amines. Toxins and Other Harmful Compounds in Foods.

[4] Karovičá J., Kohajdová Z. (2005). Biogenic Amines in Food. Chem. Pap..

[5] Ordóñez J.L., Callejón R.M., Troncoso A.M., García-Parrilla M.C. (2017). Evaluation of biogenic amines profile in opened wine bottles: Effect of storage conditions. J. Food Comp. Anal..

[6] Restuccia D., Spizzirri U.G., Puoci F., Parisi O.I., Curcio M., Picci N., Rai V.R., Bai J.A. (2014). Accumulation of Biogenic Amines in Foods: Hazard Identification and Control Options.

[7] Agudelo-Romero P., Bortolloti C., Pais M.S., Tiburcio A.F., Fortes A.M. (2013). Study of polyamines during grape ripening indicate an important role of polyamine catabolism. Plant Physiol. Biochem..

[8] Herbert P., Cabrita M.J., Ratola N., Laureano O., Alves A. (2005). Free amino acids and biogenic amines in wines and musts from the Alentejo region. Evolution of amines during alcoholic fermentation and relationship with variety, sub–region and vintage. J. Food Eng..

[9] Hernández-Orte P., Peña-Gallego A., Ibarz M.J., Cacho J., Ferreira V. (2006). Determination of the biogenic amines in musts and wines before and after malolactic fermentation using 6-aminoquinolyl-*N*-hydroxysuccinimidyl carbamate as the derivatizing agent. J. Chromatogr. A.

[10] Marcobal A., Martín-Alvarez P.J., Polo M.C., Muñoz R., Moreno-Arribas M.V. (2006). Formation of biogenic amines throughout the industrial manufacture of red wine. J. Food Proect..

[11] Rodriguez-Naranjo M.I., Ordóñez J.L., Callejón R.M., Cantos-Villar E., Garcia-Parrilla M.C. (2013). Melatonin is formed during winemaking at safe levels of biogenic amines. Food Chem. Toxicol..

[12] Romano P., Capece A., Poeta C. (2007). Biogenic amine formation in alcoholic fermentation. Bull. OIV.

[13] Wang Y.Q., Ye D.Q., Zhu B.Q., Wu G.F., Duan C.Q. (2014). Rapid HPLC analysis of amino acids and biogenic amines in wines during fermentation and evaluation of matrix effect. Food Chem..

[14] García-Marino M., Trigueros Á., Escribano-Bailón T. (2010). Influence of oenological practices on the formation of biogenic amines in quality red wines. J. Food Comp. Anal..

[15] Martuscelli M., Arfelli G., Manetta A.C., Suzzi G. (2013). Biogenic amines content as a measure of the quality of wines of Abruzzo (Italy). Food Chem..

[16] Nalazek-Rudnicka K., Wasik A. (2017). Development and validation of an LC–MS/MS method for the determination of biogenic amines in wines and beers. Monatshefte Chem..

[17] Płotka-Wasylka J., Simeonov V., Namieśnik J. (2016). An in situ derivatization—Dispersive liquid-liquid microextraction combined with gas-chromatography—Mass spectrometry for determining biogenic amines in home-made fermented alcoholic drinks. J. Chromatogr. A.

[18] Płotka-Wasylka J., Namieśnik J., Kłodzińska E. (2017). Determination of Biogenic Amines in Wine Using Micellar Electrokinetic Chromatography. J. Res. Anal..

[19] Massart D.L., Kaufman L. (1983). The Interpretation of Analytical Chemical Data by the Use of Cluster Analysis.

[20] Comuzzo P., Rauhut D., Werner M., Lagazio C., Zironi R. (2013). A survey on wines from organic viticulture from different European countries. Food Control.

[21] Ramos R.M., Valente I.M., Rodrigues J.A. (2014). Analysis of biogenic amines in wines by salting-out assisted liquid-liquid extraction and high-performance liquid chromatography with fluorimetric detection. Talanta.

