# Supplementary material for: Evaluation of the Impact of Storage Conditions on the Biogenic Amines Profile in Opened Wine Bottles
Source: Molecules. 2018 May 9;23(5):1130. doi: 10.3390/molecules23051130 (PMC6100326; doi:10.3390/molecules23051130)
Supplement: Supplementary file 1 [file molecules-23-01130-s001.pdf]

Article

Evaluation of storage conditions impact on biogenic amines profile in opened wine bottles

Justyna Płotka-Wasylka [1],*, Vasil Simeonov [2] and Jacek Namieśnik [1]

[1] Department of Analytical Chemistry, Faculty of Chemistry, Gdańsk University of Technology, 11/12 Narutowicza Street, 80-233 Gdańsk, Poland; e-mail:

[2] Faculty of Chemistry and Pharmacy, University of Sofia, 1 James Bourchier Blvd., Sofia 1126, Bulgaria; vsimeonov@chem.uni-sofia.bg

* Correspondence: plotkajustyna@gmail.com; juswasyl@pg.edu.pl; Tel.: +48 58 347 21 10

Received: date; Accepted: date; Published: date

Abstract: A survey of biogenic amine (BA) profiles in opened wine bottles has been established to monitor the level of biogenic amines (BAs) in opened bottles against time and other conditions. Bottles of red and white wine were submitted to different temperatures, stopper type (screw cap, cork), and use of vacuum devices. A total of six wines made from a variety of grapes were obtained from vineyards from regions across Poland. Dispersive liquid-liquid microextraction-gas chromatography-mass spectrometry (DLLME-GC-MS) procedure for BAs determination was validated and applied for wine sample analysis. The total content of BAs from the set of immediately opened wine samples ranged from 442 to 929 μg/L for white wines, and 669 to 2244 μg/L for red wines. The most abundant BAs in the analysed wines were histamine and putrescine. Considering the commercial availability of the analysed wines, there was no relationship between the presence of BAs in a given wine and their availability on the market. However, it was observed and confirmed by chemometric analysis that the different storage conditions employed in this experiment affect not only the BAs profile, but also the pH.

Keywords: Biogenic amines; chemometric analysis; DLLME, GC-MS; storage conditions; stopper type

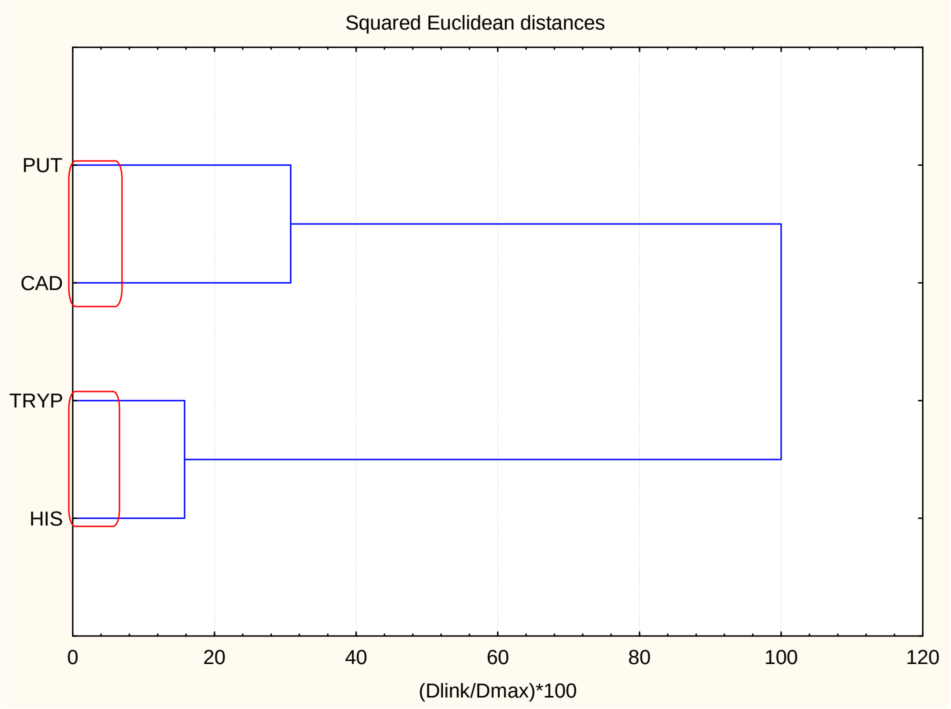

Figure 1 SI. Variable clustering for Wine B

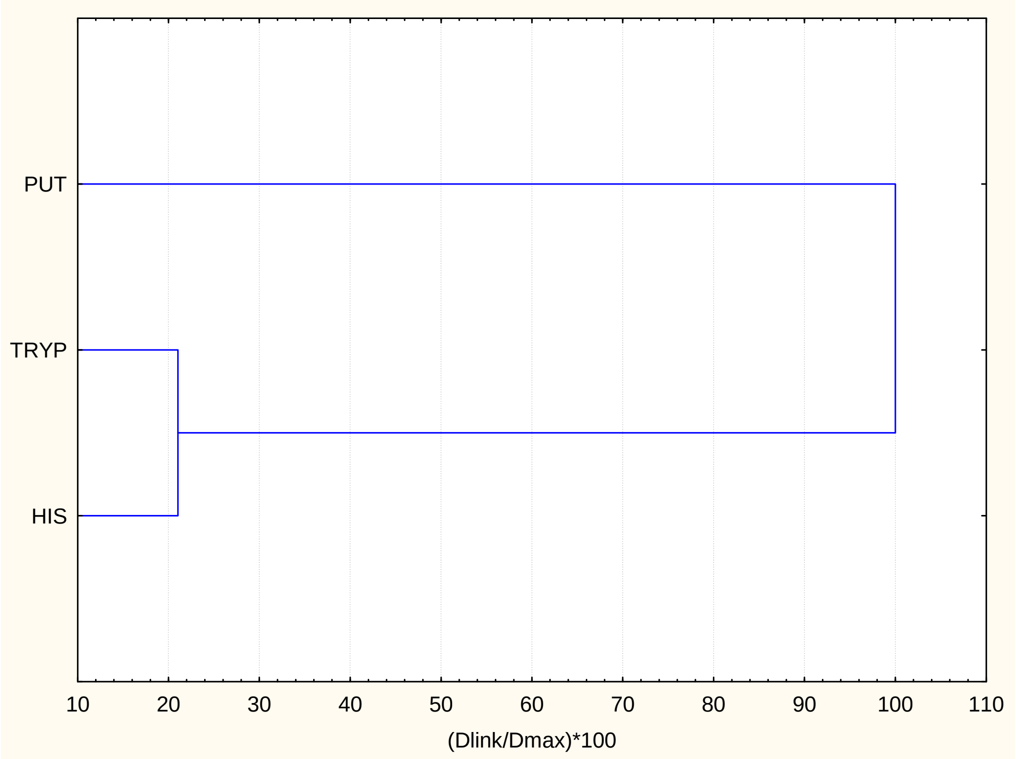

Figure 2 SI. Variable clustering for Wine C

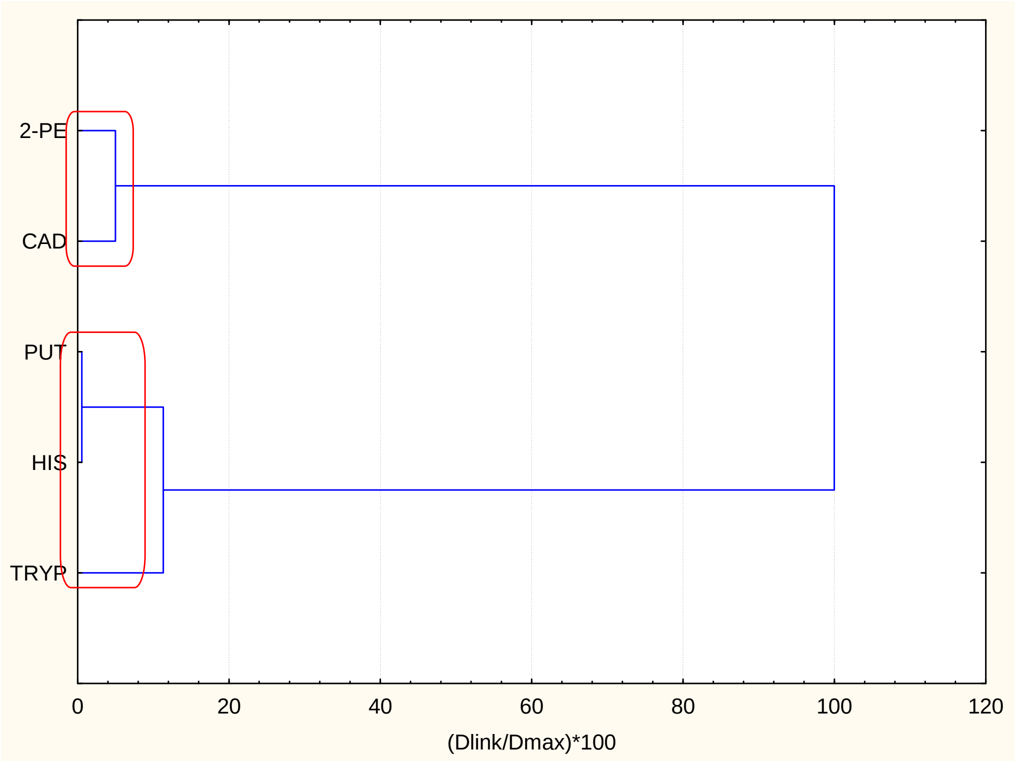

Figure 3 SI. Variable clustering for Wine D

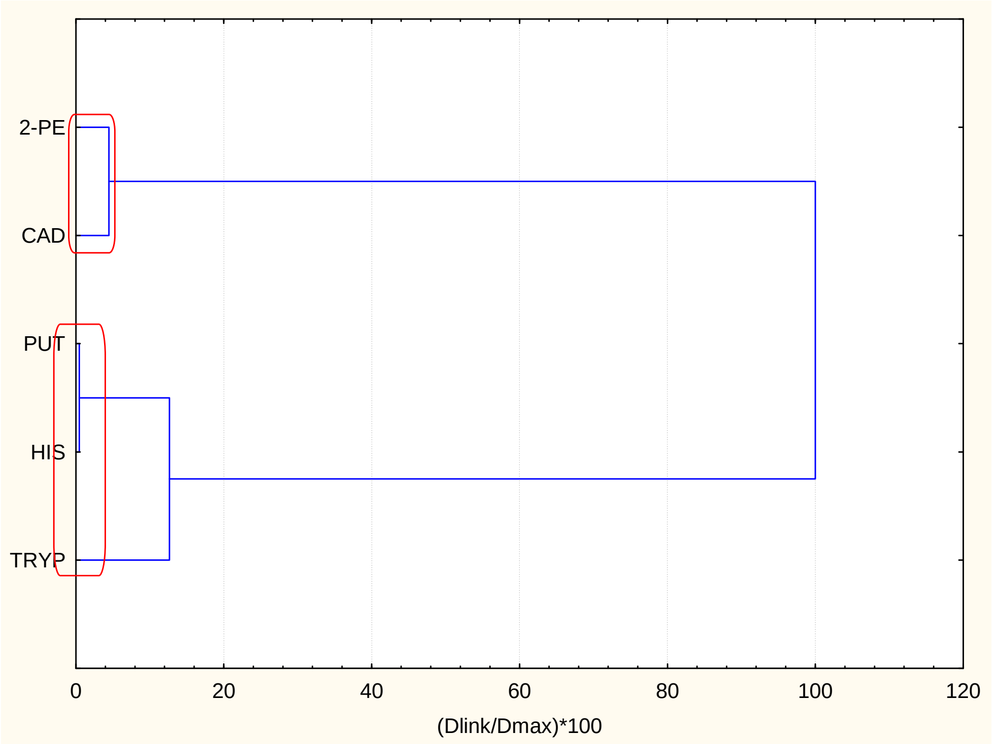

Figure 4 SI. Variable clustering for Wine E

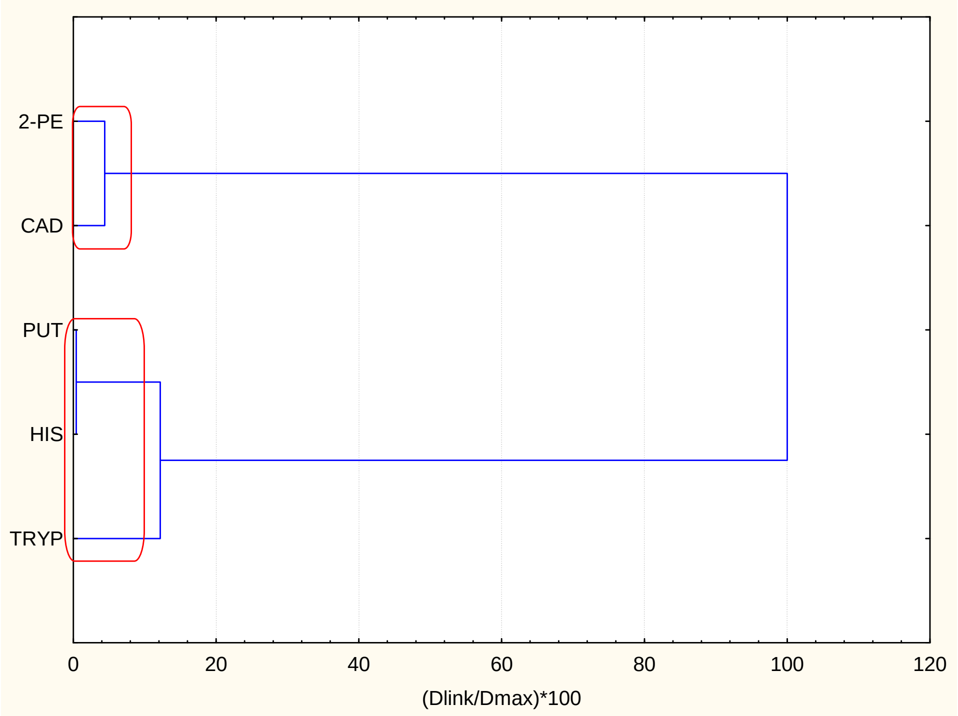

Figure 5 SI. Variable clustering for Wine F

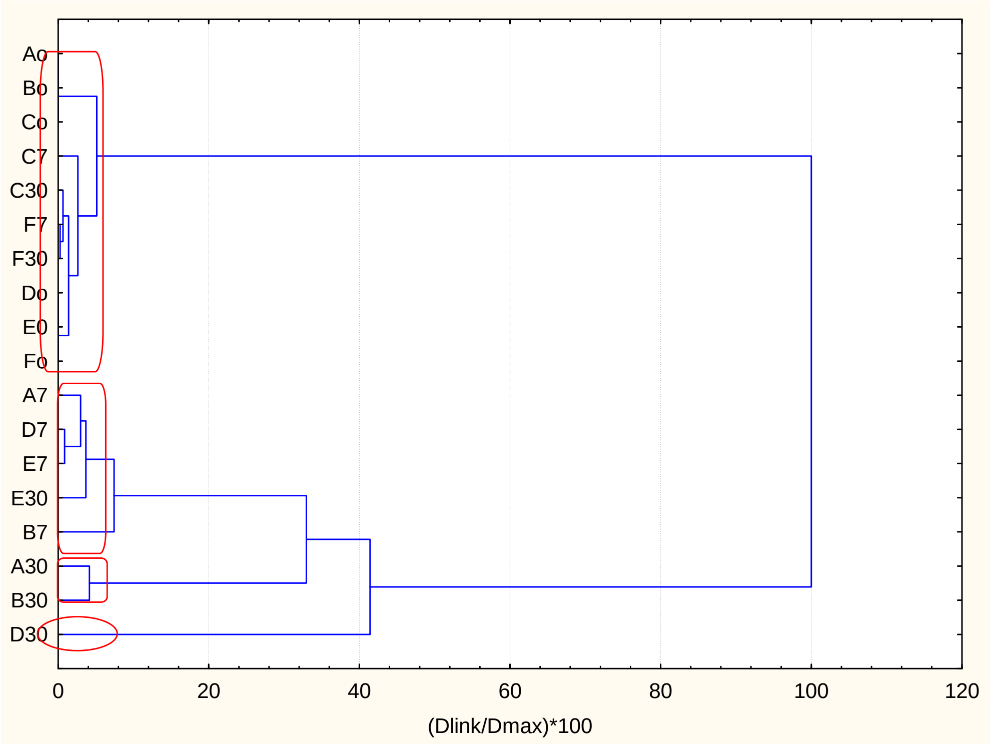

Figure 6 SI. Hierarchical dendrogram for wine samples (Wine B)

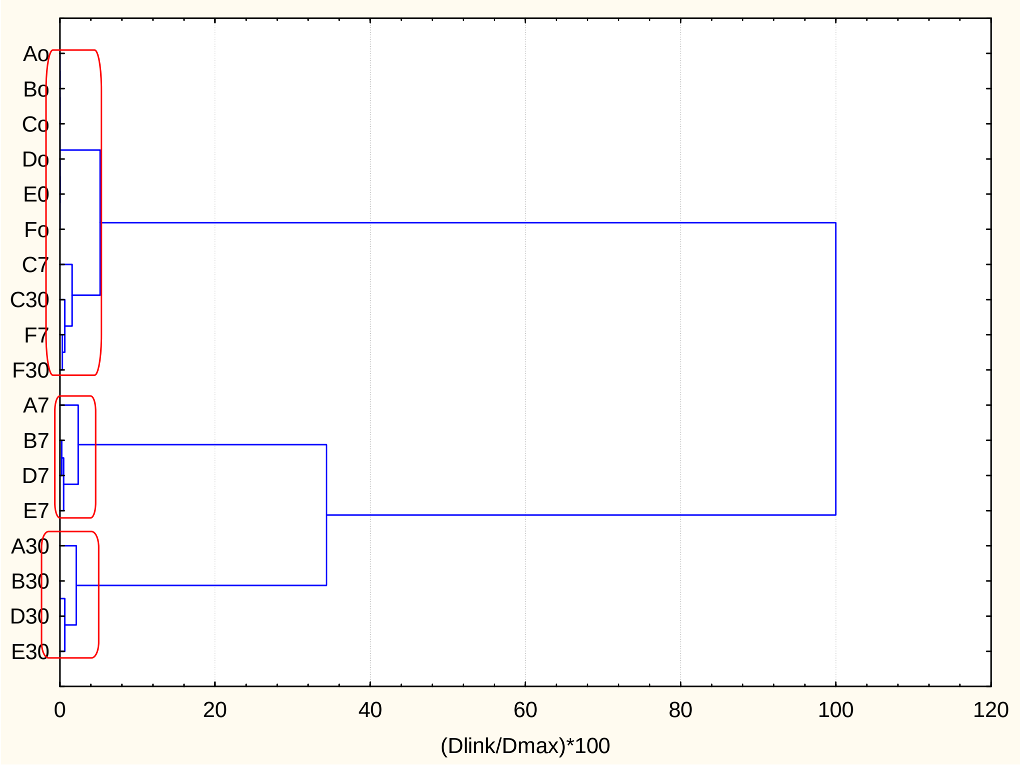

Figure 7 SI. Hierarchical dendrogram for wine samples (Wine C)

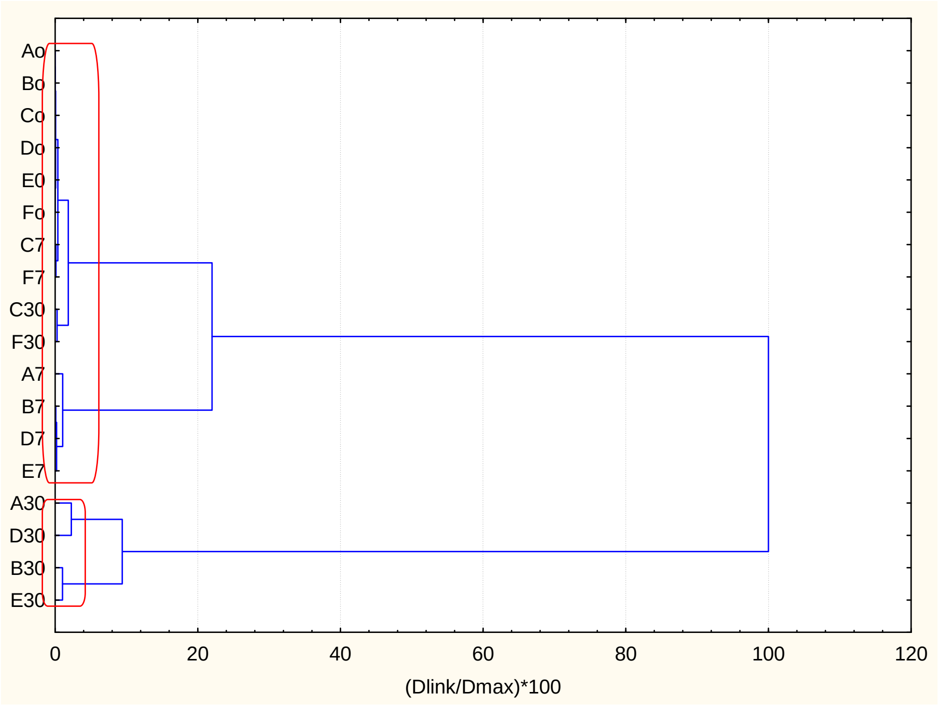

Figure 8 SI. Hierarchical dendrogram for wine samples (Wine D)

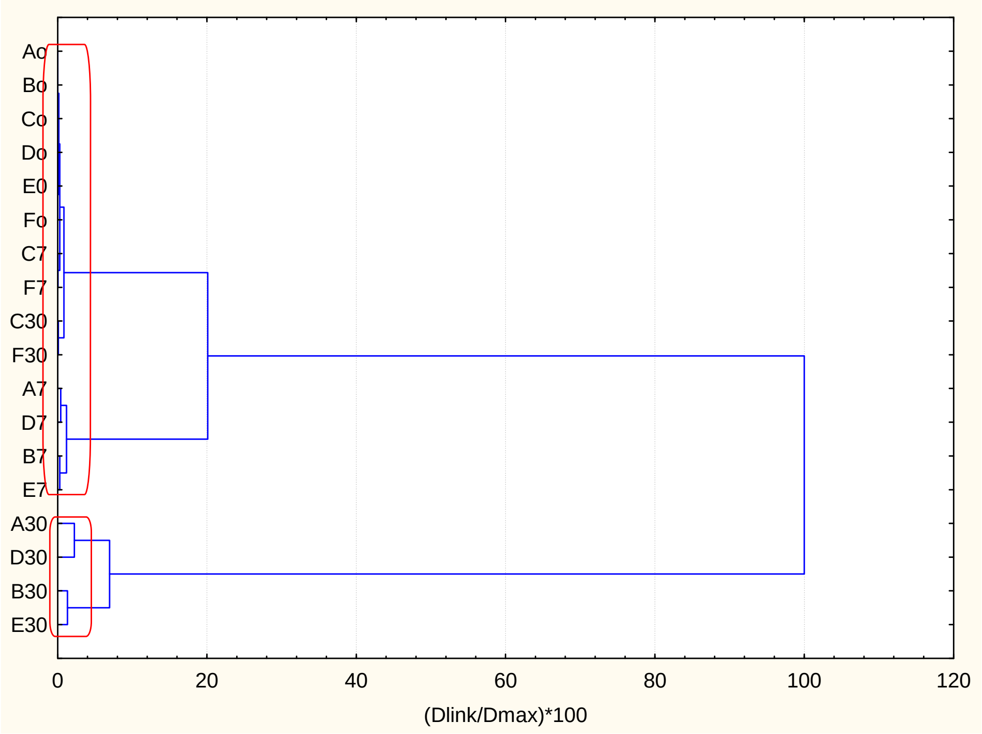

Figure 9 SI. Hierarchical dendrogram for wine samples (Wine E).

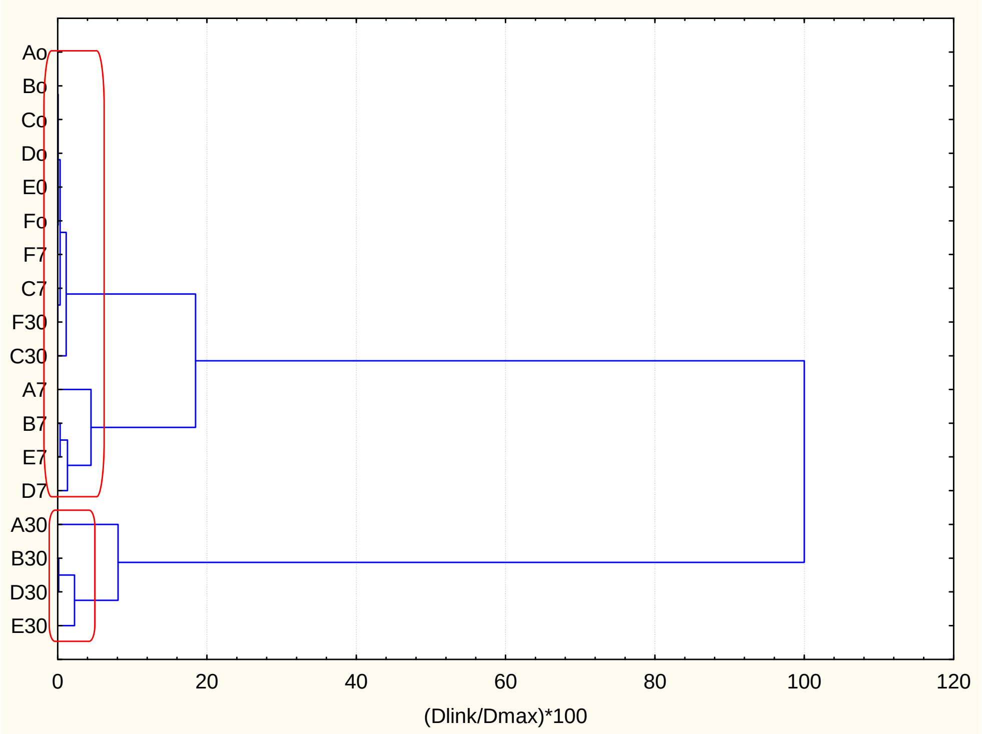

Figure 10 SI. Hierarchical dendrogram for wine samples (Wine F)
